# Physalin A Inhibits MAPK and NF-κB Signal Transduction Through Integrin αVβ3 and Exerts Chondroprotective Effect

**DOI:** 10.3389/fphar.2021.761922

**Published:** 2021-12-01

**Authors:** Rui Lu, Xiaojun Yu, Shuang Liang, Peng Cheng, Zhenggang Wang, Zhi-yi He, Zheng-tao Lv, Junlai Wan, Haokun Mo, Wen-tao Zhu, An-min Chen

**Affiliations:** ^1^ Department of Orthopedics, Tongji Hospital, Tongji Medical College, Huazhong University of Science and Technology, Wuhan, China; ^2^ Department of Physiology and Pharmacology, Karolinska Institutet, Stockholm, Sweden

**Keywords:** physalin A, osteoarthritis, αvβ3, integrin, MAPK, NF-κB

## Abstract

Osteoarthritis (OA) is a common articular ailment presented with cartilage loss and destruction that is common observed in the elderly population. Physalin A (PA), a natural bioactive withanolide, exerts anti-inflammatory residences in more than a few diseases; however, little is known about its efficacy for OA treatment. Here, we explored the therapeutic effects and potential mechanism of PA in mouse OA. After the *in vitro* administration of PA, the expression of inflammation indicators including inducible nitric oxide synthase and cyclooxygenase-2 was low, indicating that PA could alleviate the IL-1β-induced chondrocyte inflammation response. Moreover, PA reduced IL-1β-induced destruction of the extracellular matrix by upregulating the gene expression of anabolism factors, including collagen II, aggrecan, and sry-box transcription factor 9, and downregulating the gene expression of catabolic factors, including thrombospondin motif 5 and matrix metalloproteinases. In addition, the chondroprotective effect of PA was credited to the inhibition of mitogen-activated protein kinase (MAPK) and nuclear factor-κB (NF-κB) signaling pathways. Furthermore, *in vivo* experiments showed that intra-articular injection of PA could alleviate cartilage destruction in a mouse OA model. However, the anti-inflammatory, anabolism enhancing, catabolism inhibiting, and MAPK and NF-κB signaling pathway inhibiting properties of PA on IL-1β-induced chondrocytes could be reversed when integrin αVβ3 is knocked down by siRNA. In conclusion, our work demonstrates that PA exhibits a chondroprotective effect that may be mediated by integrin αVβ3. Thus, PA or integrin αVβ3 might be a promising agent or molecular target for the treatment of OA.

## Introduction

OA is the most common articular disorder in the world, affecting 9.6% of men and 18% of women over 60, and is characterized by cartilage damage, joint space narrowing, sclerosis or osteoporosis of the subchondral bone, formation of osteophytes, and synovial membrane inflammation, which occurs in weight-bearing joints and joints subjected to high levels of activity. Common symptoms of OA include the slow development of pain, stiffness, swelling, constrained movement, and deformity of the affected joint, and the pain and dysfunction caused by this disease are the most important factors impacting the lives of elderly people (Woolf and Pfleger, 2003). It is also a degenerative disease influenced by aging, trauma, obesity, strain, joint deformity, and many other factors; however, the exact mechanism of OA remains unclear (Glyn-Jones et al., 2015; Jeon et al., 2017).

According to a previous study, inflammation is regarded as one of the leading pathogenic factors in the progression of OA (Pelletier et al., 2001). Thus, an imbalance of pro-inflammatory and anti-inflammatory factors in the body has an important effect on OA, as these two factors are essential for maintaining a steady state of catabolism and anabolism in articular cartilage (Lee et al., 2021). Interleukin 1β (IL-1β), which is an important pro-inflammatory cytokine mediating the pathophysiology of OA, plays a key role in weakening chondrocyte anabolism and improving chondrocyte catabolism (Liacini et al., 2002). IL-1β can also trigger high synthesis of catabolic index proteins including thrombospondin motifs (ADAMTS) and matrix metalloproteinases (MMPs) and reduce the expression of anabolic proteins like collagen II and aggrecan (Hosseinzadeh et al., 2016). Moreover, inhibiting IL-1β could counteract the disordered metabolic process of OA (Kapoor et al., 2011; Appleton, 2018; Lin et al., 2021). Therefore, anti-inflammatory therapy is pivotal during the development of OA, with drugs for anti-inflammatory routinely applied to relieve patient symptoms (Petersen et al., 2019). However, these drugs and methods are not effective at reducing or reversing the symptoms of OA (Crowley et al., 2009).

PA is a natural acid slurry extracted from Physalis alkekengi L. var. franchetii (Mast.) Makino that has many pharmacological functions, such as anti-inflammatory (Lin et al., 2020; Wang et al., 2021b), anti-tumor (Han et al., 2011; He et al., 2013a; He et al., 2013b; He et al., 2014; Kang et al., 2016), anti-fungal, anti-cough, and analgesic activities. The MAPK, together with NF-κB signaling pathway, is thought to play crucial roles in the development of OA, as activation of these two pathway causes cartilage inflammation and damage (Lianxu et al., 2006; Lepetsos et al., 2019; Chuntakaruk et al., 2021; Deng et al., 2021). Therefore, inhibition of these pathways may be a method of OA treatment (Wu et al., 2020). PA regulates molecular mechanism of inflammation, proliferation, autophagy, and apoptosis by mediating the MAPK (Kang et al., 2016; Shin et al., 2019), NF-κB (He et al., 2013a), and reactive oxygen species (Kang et al., 2016) signaling pathways under different pathophysiological conditions. However, research about the effect of PA on OA chondrocytes and cartilage has not been implemented to date. Therefore, this study explores the role of the anti-inflammatory or signaling pathway-suppressing effects of PA in the treatment of mice OA and elucidates the potential molecular mechanisms.

## Materials and Methods

### Chemicals and Reagents

**FIGURE 1:**
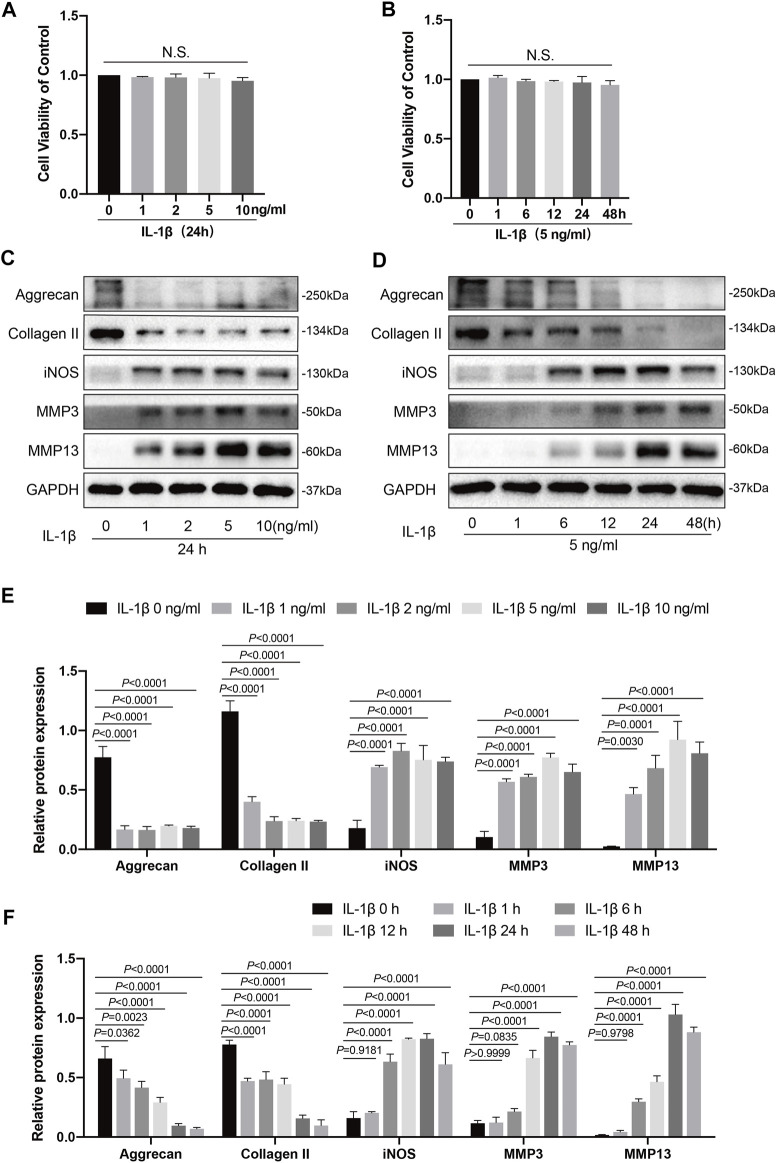
Effects of IL-1β on knee chondrocytes in mice. **(A)** Mice chondrocytes were treated with various concentration gradients of IL-1β (0, 1, 2, 5, and 10 ng/ml) for 24 h, **(B)** or with IL-1β at the concentration of 5 ng/ml for different time points (0, 1, 6, 12, 24, and 48 h), and cell viability was detected with a CCK-8 kit. **(C)** Western blots and **(E)** quantitative analysis of aggrecan, collagen II, iNOS, MMP3, and MMP13 for chondrocytes exposed to different concentrations of IL-1β (0, 1, 2, 5, and 10 ng/ml) for 24 h. **(D)** Western blotting results and **(F)** quantitative analysis of aggrecan, collagen II, iNOS, MMP3, and MMP13 for chondrocytes stimulated with IL-1β (5 ng/ml) at several time points (0, 1, 6, 12, 24, and 48 h). GAPDH was used as an internal reference. Data are presented as means ± SD (*n* = 3). The exact *p* value was marked in the corresponding figure, N.S. indicated no significance, and *p* < 0.05 was considered statistically significant.

**FIGURE 2:**
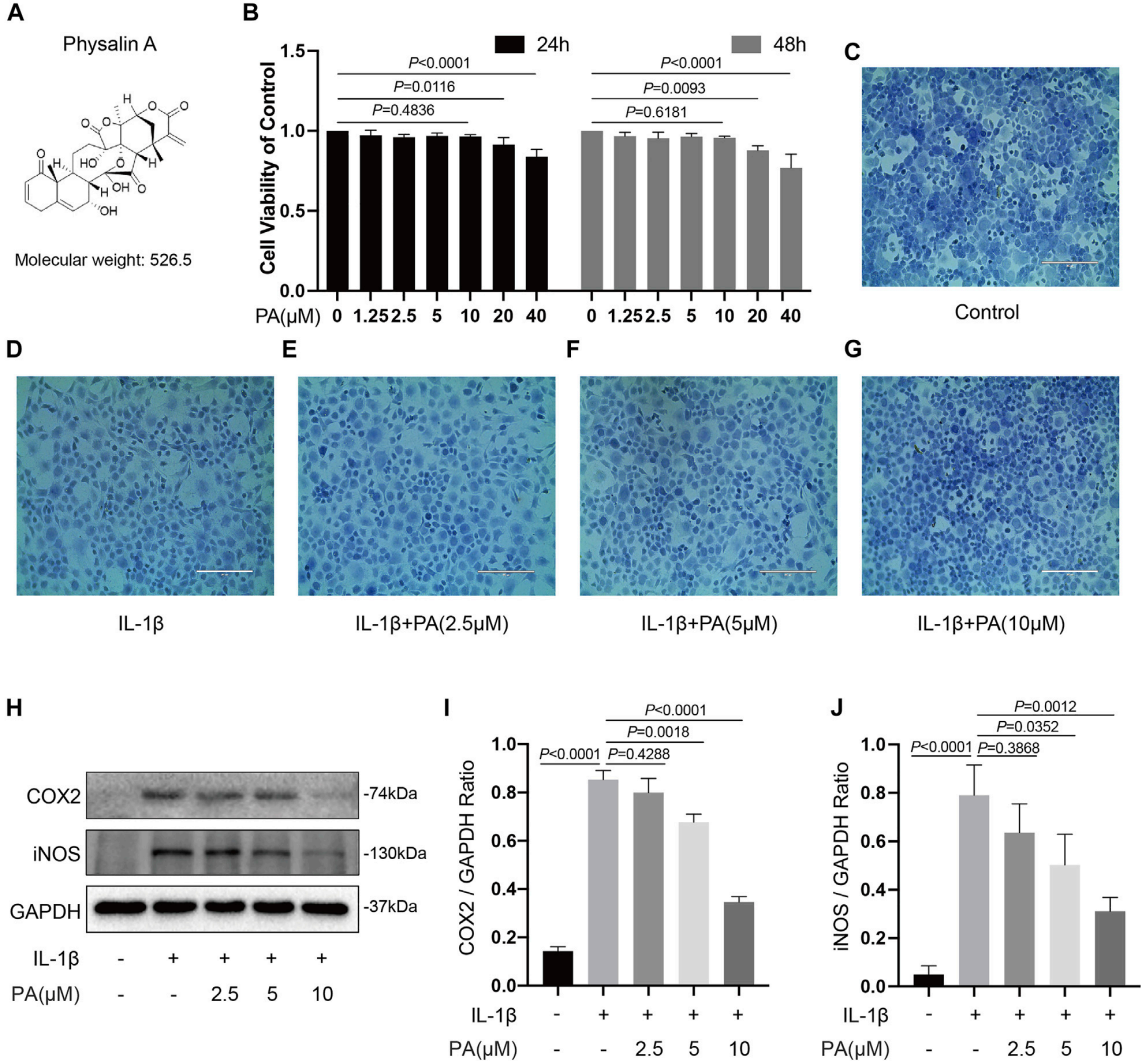
Effects of PA on cell viability and PA suppressed inflammatory responses induced by IL-1β. **(A)** Molecular structure of PA. **(B)** Mice chondrocytes were treated with PA (0, 1.25, 2.5, 5, 10, 20, and 40 μM) for 24 and 48 h, and cell viability was detected with a CCK-8 kit. **(C–G)** Toluidine blue staining of chondrocytes treated with IL-1β (5 ng/ml) and PA for 24 h (scale bar 200 μm). **(H)** Western blotting results and **(I,J)** quantitative analysis of COX2 and iNOS in IL-1β–induced chondrocytes treated with PA. GAPDH was used as an internal reference. Data are presented as means ± SD (*n* = 3). The exact *p* value was marked in the corresponding figure and *p* < 0.05 was considered statistically significant.

**FIGURE 3:**
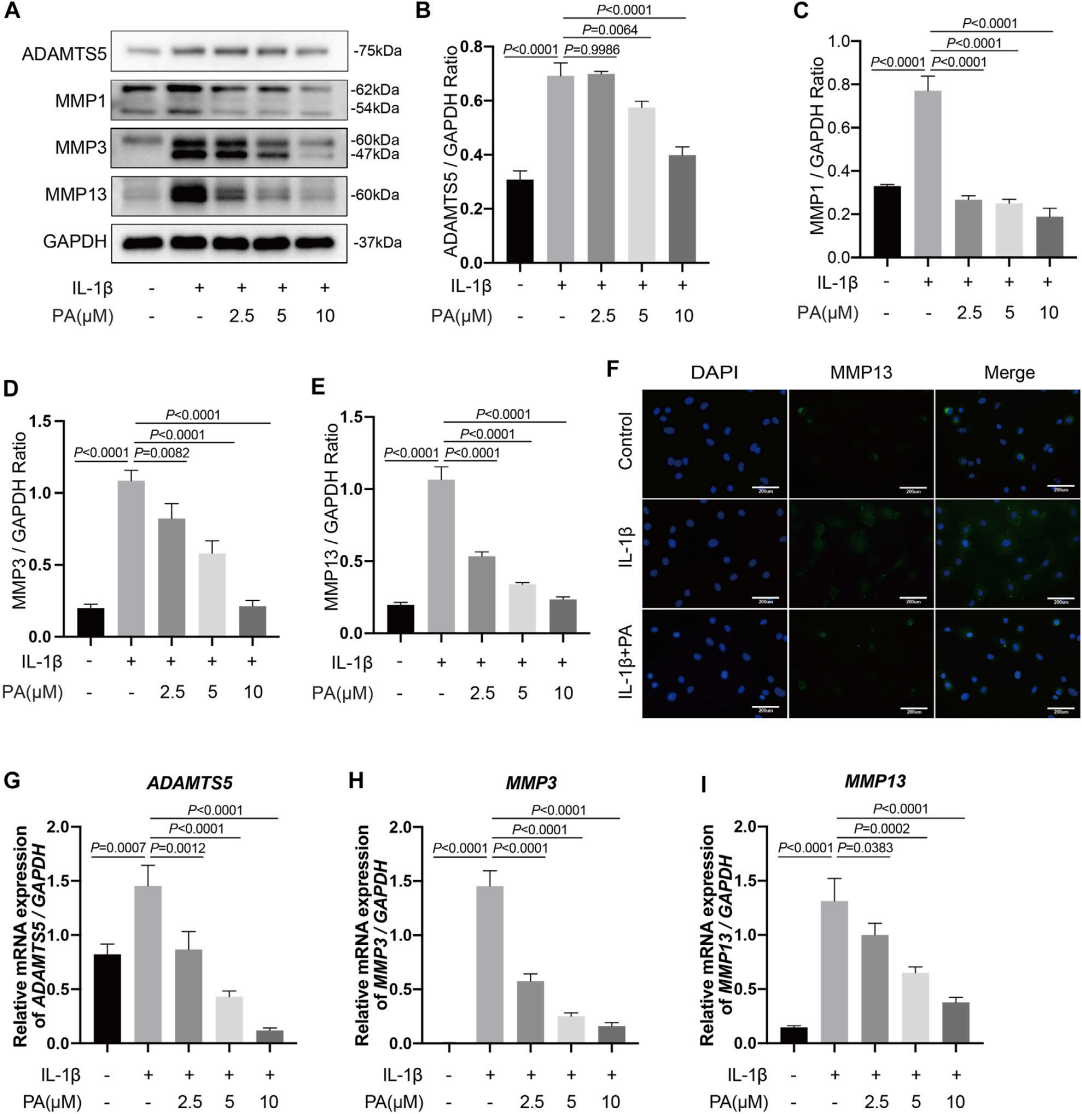
PA suppressed excess expression of the catabolic indicators of chondrocytes induced by IL-1β, including ADAMTS5, MMP1, MMP3, and MMP13. Mice chondrocytes were treated with 5 ng/ml of IL-1β, alone or with PA (2.5, 5, and 10 μM) for 24 h **(A)** Western blotting results and **(B–E)** quantitative analysis of ADAMTS5, MMP1, MMP3, and MMP13. **(F)** MMP13 expression was observed by immunofluorescence staining when chondrocytes were treated with 5 ng/ml of IL-1β, alone or with 10 μM of PA (scale bar 200 μm). **(G–I)** Relative mRNA levels of ADAMTS5, MMP3, and MMP13 in chondrocytes stimulated with 5 ng/ml of IL-1β, alone or with PA (2.5, 5, and 10 μM) for 24 h. GAPDH was used as an internal reference. Data are presented as means ± SD (*n* = 3). The exact *p* value was marked in the corresponding figure and *p* < 0.05 was considered statistically significant.

Physalin A (CAS Registry Number: 23027-91-0) was purchased from ChemFaces (Wuhan, Hubei, China), the molecular structure can be found in Figure 2A. Safranin O solution (Catalog number: G1067) and toluidine blue (Catalog number: G3660) were provided by Solarbio (Beijing, China). The company R&D Systems (Minneapolis, MN, United States) provided cytokine IL-1β for mouse. Primary antibody against ADAMTS5 (A02802-1) was got from Boster (Wuhan, Hubei, China) and was applied at a 1:500 dilution. Primary anti-MMP3 antibody (ab52915, applied in Figure 1) was obtained from Abcam (Cambridge, United Kingdom) and used at a 1:1,000 dilution. The biotechnology company Proteintech Group (Wuhan, Hubei, China) supplied the primary anti-GAPDH (60004-1-Ig) and anti-MMP3 (66338-1-Ig, used in Figure 3 and Figure 6) antibodies which were used at a 1:10,000 and 1:5,000 dilution respectively, primary anti-MMP1 (10371-2-AP), anti-MMP13 (18165-1-AP), anti-aggrecan (13880-1-AP), anti-collagen II (28459-1-AP) antibodies were used at a 1:1,000 dilution. The biotech company CST (Beverly, MA, United States) provided the primary antibodies including anti-SOX9 (#82630), anti-iNOS (#13120), anti-COX-2 (#12282), anti-P-P38 (#4511), anti-P38 (#8690), anti-p-JNK (#4668), anti-JNK (#9252), anti-p-ERK (#4370), anti-ERK (#4695), anti-P-P65 (#3033), anti-P65 (#8242), and all used at a dilution ratio of 1:1,000. Anti-rabbit and anti-mouse secondary antibodies for western blot and immunofluorescence analyses, phosphate buffer saline (PBS) solution, tyrisin, and collagenase type II were acquired from Boster (Wuhan, Hubei, China).

### Extraction and Culture of Chondrocytes

C57BL/6 mice (5 days old, male) were sacrificed to obtain chondrocytes according to a reported method (Li et al., 2018). In short, the knee cartilage was extracted, then cut into tiny granules and incubated with 0.25% trypsin-EDTA in a cell incubator for 0.5 h. After centrifugation, the 0.25% trypsin-EDTA was removed and 0.2% collagenase type II was utilized to digested the cartilage granules for 6–8 h at 37°C in a hybridization oven. Collagenase type II was removed after centrifugation, and cartilage cells were suspended and maintained in a medium containing 10% fetal bovine serum at a temperature of 37°C and a CO_2_ concentration of 5%. Chondrocytes from the first passage were selected for subsequent experiments.

### Cell Viability

The viability of chondrocytes pretreated with PA was evaluated by cell counting kit-8 (CCK-8). Cells in 96-well plates (5,000–10,000 cells/well) were cultured for 24 h. The CCK-8 assay reagent was not added into the wells (10 µL/well) until cells were processed with various concentration gradients of IL-1β (0, 1, 2, 5, and 10 ng/ml) for 24 h, or with IL-1β at a concentration of 5 ng/ml for different time points (0, 1, 6, 12, 24, and 48 h), or with various concentration gradients of PA (0, 1.25, 2.5, 5, 10, 20, and 40 μM) for 24 and 48 h. Next, the absorbance of chondrocytes was obtained using a microplate reader (Bio-Rad, Richmond, United States) to reflect the influence of PA on the growth of chondrocytes.

### Toluidine Blue Staining

Toluidine blue staining was applied to reveal the morphology of cartilage cells treated with PA of different concentrations and IL-1β (5 ng/ml), according to a previously described method (Shepard and Mitchell, 1976; Schmitz et al., 2010). When chondrocytes reached 80% confluency, different concentrations of PA alone or with IL-1β (5 ng/ml), were added to treat the cells. The cells then continued to incubate for 24 h. Subsequently, cells were washed with PBS (three times, 5 min each) before fixation with 4% paraformaldehyde (15 min) at 25°C. After discarded the cell fixative and wash the cells (three times, 5 min each), toluidine blue dye was added into the wells and the chondrocytes were stained with the reagent for 2 h. Finally, the toluidine blue liquid was eliminated and chondrocytes were washed again. Images of chondrocyte morphology were captured by a microscope (Evos Fl Auto, Life Technologies, United States).

### Safranin O Staining

According to a previous study, safranin O reagent could reflect the content of proteoglycan (Schmitz et al., 2010). The procedure for safranin O staining was similar to that of toluidine blue staining. Briefly, when chondrocytes reached 80% confluency in a 24-well plate, different concentrations of PA alone or with IL-1β (5 ng/ml), were added into each well. The cells then continued to incubate for 24 h before being washed with PBS, fixed with paraformaldehyde at 25°C, discarded the cell fixative, and stained with safranin O reagent for 2 h. At last, the safranin O dye was eliminated and the chondrocytes were again washed with tri-distilled water. The aforementioned microscope was used to observe the proteoglycan content of the chondrocytes with different interventions.

### Western Blotting Analysis

Protein samples was gained from chondrocytes with different treatment. In brief, chondrocytes were collected after lysis on ice for 15 min with the cell lysate, which was formulated in a proportion of 100:1:1 with the RIPA lysis buffer, phosphatase inhibitors, and protease inhibitors (Boster, Wuhan, China). Cells were further lysed with an ultrasonic disruptor. The supernatant after centrifugation was then collected as protein samples. After the detection of samples concentration with the above mentioned reader, the proteins were mixed thoroughly with a protein loading buffer, cooked at 100°C for 5 min, then stored at −20°C for follow-up experiments. Electrophoresis and membrane transferred were performed after the protein samples were loaded onto SDS-PAGE gels (8.0–12.5%). After 1 h of blocking with 5% BSA, the bands were incubated with primary antibodies at 4°C overnight. Next, the samples were rinsed with TBST (three times, 10 min each) and incubated with the corresponding proportion of secondary antibodies for 1 h at 25°C, then washed again with TBST (three times, 10 min each). Finally, a exposure software was applied to visualize the target protein bands.

### Quantitative Real-Time Polymerase Chain Reaction

Relevant indicators of the anabolism and catabolism of chondrocytes at the gene level were evaluated with RT-qPCR analysis. Briefly, a kit for RNA extraction (Omega Bio-tek, United States) was utilized to obtain the total RNA of chondrocytes. The quality of the total RNA samples was verified by the aforementioned microplate reader. The total RNA samples and a kit for complementary DNA (cDNA) synthesis were used to synthesize cDNA, which was subsequently amplified using a RT-qPCR kit (Yeasen, Shanghai, China). The relative levels of different gene expressions were represented as bar graphs by calculating the comparative2^−ΔΔCt^. And primer sequences used in the experiment are shown in Table 1.

**TABLE 1:** Primer sequence used in the RT-qPCR experiment.

Gene	Sequence
*MMP3*	Forward: 5′-ACT​CCC​TGG​GAC​TCT​ACC​AC-3′
	Reverse: 5′-GGT​ACC​ACG​AGG​ACA​TCA​GG-3′
*MMP13*	Forward: 5′-TGA​TGG​ACC​TTC​TGG​TCT​TCT​GG-3′
	Reverse: 5′-CAT​CCA​CAT​GGT​TGG​GAA​GTT​CT-3′
*ADAMTS5*	Forward: 5′-CCC​AGG​ATA​AAA​CCA​GGC​AG-3′
	Reverse: 5′-CGG​CCA​AGG​GTT​GTA​AAT​GG-3′
*Collagen II*	Forward: 5′-GGC​CAG​GAT​GCC​CGA​AAA​TTA-3′
	Reverse: 5′-CGC​ACC​CTT​TTC​TCC​CTT​GT-3′
*Aggrecan*	Forward: 5′-AGG​TGT​CGC​TCC​CCA​ACT​AT-3′
	Reverse: 5′-CTT​CAC​AGC​GGT​AGA​TCC​CAG-3′
*SOX9*	Forward: 5′-AGG​AAG​TCG​GTG​AAG​AAC​GG-3′
	Reverse: 5′-GGA​CCC​TGA​GAT​TGC​CCA​GA-3′
*Itg αV*	Forward: 5′-AAT​AAG​ATC​TGC​CCG​TTG​CC-3′
	Reverse: 5′-GTA​GAA​GCT​CCA​CCT​GGA​AG-3′
*Itg β3*	Forward: 5′-TGA​TGG​GCA​CTG​TCA​CAT​TG-3′
	Reverse: 5′-TTC​TGG​TAA​AGG​CTG​ACG​AC-3′
*GAPDH*	Forward: 5′-CCC​AGC​TTA​GGT​TCA​TCA​GG-3′
	Reverse: 5′-ATC​TCC​ACT​TTG​CCA​CTG​C-3′

### Immunofluorescence

Chondrocytes were not processed with PA (10 μM) alone or with IL-1β (5 ng/ml) in a 24-well plate (10,000 cells/well) for 24 h until the cells reached 40% confluency. Next, chondrocytes were pretreated with 4% paraformaldehyde and 0.2% Triton X-100 for 15 min respectively. After blocked with 5% BSA for 30 min, the chondrocytes were incubated overnight with primary rabbit antibodies against MMP13 (18165-1-AP, 1:50), collagen II (28459-1-AP, 1:200), aggrecan (13880-1-AP, 1:200), and P65 (#8242, 1:400) at 4°C. The chondrocytes were then incubated with the corresponding species of secondary antibody (1:50 dilution) in the absence of light at 37°C for 1 h. Under dark conditions, the cells were immersed with 4–6-Diamidino-2-phenylindole dye (DAPI) for 10 min. Then, immunofluorescence images were obtained by the aforementioned microscope.

### siRNA

Small interfering RNA (siRNA) was selected to knock down specific genes. The siRNA (sequence:5′-GAGGATCTCTTCAACTCTA-3′, 5′-CCG​TGA​ATT​GTA​CCT​ACA​A-3′), synthesized by RiboBio (Guangzhou, China), was used to target the mouse integrins αV (Itg αV) and β3 (Itg β3), respectively. The specific integrin αVβ3 siRNA was used to transfect chondrocytes. After reaching 60–70% confluency in a six-well plate, the chondrocytes were incubated with negative control or integrin αVβ3 siRNA (transfection concentration, 50 nM) for 24 h with the help of lipofectamine 3,000 reagent for transfection (Thermo Fisher, UT, United States). Subsequently, the knockdown efficiency was verified by RT-qPCR, and the cells transfected by the above method were cultured for further treatment.

### Surgical Procedures for DMM-OA Models

The male mice of C57BL/6J, which were bred in the specific-pathogen-free Animal Center of Tongji Medical College, Huazhong University of Science and Technology, served as the experimental OA models. After anesthetization with 1% pentobarbital (100 µl/10 g body weight, intraperitoneal administration), 24 mice were randomly selected and grouped (the DMM group and the DMM + PA group) for destabilized medial meniscus (DMM) surgery. The remaining 12 mice underwent only capsulotomy and suturing surgery (the sham group). All surgical procedures were performed only on the right knee of the mice. Three groups were administered intra-articular injections with different treatments, twice per week. The sham and DMM groups received 10 μl of the vehicle (30% PEG300, 5% DMSO, and ddH_2_O), whereas the DMM + PA group received 10 μl of PA (1 mg/kg body weight).

### Histological Staining and Immunohistochemistry Analysis

After 8 weeks of intra-articular injection, the right knee joints of all mice were obtained. After immersion in tissue fixative for 24 h, the knee joints were transferred into regent containing 10% EDTA for decalcification for 30 days. Next, these decalcified samples were paraffin-embedded and cut into 5-μm sections for further tissue staining analysis, which included hematoxylin-eosin (HE), safranin O/fast green. Immunohistological analysis was performed using antibodies against MMP13 (18165-1-AP, 1:100), aggrecan (13880-1-AP, 1:200) and collagen II (28459-1-AP, 1:800). The severity of OA was assessed in a blinded manner by three independent observers under the guidelines of the Osteoarthritis Research Society International (OARSI) scoring system.

### Statistical Analysis

The results, which were repeated at least three times in our experiments, were processed with GraphPad Prism V.8.4.0 software. Data were shown as the mean ± standard deviation (SD) and analyzed by one-way analysis of variance (ANOVA). Besides, the nonparametric data (OARSI scores) was analyzed by the Kruskal-Wallis H test. Statistical significance was defined as *p*-value < 0.05.

## Results

### IL-1β Inhibited Anabolism and Enhanced the Inflammatory Response and Catabolism of Chondrocytes

IL-1β is a critical pro-inflammatory mediator that inhibits chondrocyte anabolism and promotes chondrocyte catabolism, which could affect the homeostasis of chondrocytes and accelerate cartilage degradation (Kapoor et al., 2011). The viability of chondrocytes administered with various concentration gradients of IL-1β (0, 1, 2, 5, and 10 ng/ml) for 24 h, or with IL-1β at a concentration of 5 ng/ml for different time points (0, 1, 6, 12, 24, and 48 h) was measured using a CCK-8 kit. As shown in Figures 1A,B, different concentration gradients of IL-1β (0, 1, 2, 5, and 10 ng/ml) for 24 h, or IL-1β at the concentration of 5 ng/ml for different time points (0, 1, 6, 12, 24, and 48 h) exhibited no cytotoxicity on chondrocytes. Therefore, we then chose concentration gradients (0, 1, 2, 5, and 10 ng/ml) of IL-1β to clarify its effects on the inflammation response, catabolism, and anabolism of chondrocytes by western blotting. After stimulation with IL-1β at different concentrations for 24 h, the levels of inflammatory markers (iNOS) and catabolic markers (MMP3 and MMP13) were increased from those of the control group (0 ng/ml); this increase was concentration dependent and reached a peak at 5 ng/ml for MMP3 and MMP13 (Figures 1C,E). Meanwhile, the expression levels of anabolic indicators (collagen II and aggrecan) showed a decreasing trend compared to the control group (0 ng/ml). In addition, no statistical significance (*p >* 0.05) was observed in the production of collagen II, aggrecan, iNOS, or MMP13 among the groups administered with 2, 5, and 10 ng/ml of IL-1β (Figures 1C,E). Thus, we chose a IL-1β concentration of 5 ng/ml for the follow-up experiment. Next, we tested several time points (0, 1, 6, 12, 24, and 48 h) to determine the influence of IL-1β stimulation (5 ng/ml) on the inflammatory response, catabolism, and anabolism of chondrocytes. As illustrated in Figures 1D,F, changes in the inflammatory response, anabolism, and catabolism of chondrocytes stimulated with IL-1β (5 ng/ml) were generally time dependent, unlike the control group (0 h). In detail, anabolic indicators (collagen II and aggrecan) showed a downward trend, whereas inflammatory markers (iNOS) and catabolic markers (MMP3 and MMP13) showed an upward trend. Additionally, collagen II and aggrecan reached their lowest levels at 48 h, whereas iNOS, MMP3, and MMP13 peaked at 24 h. Therefore, we selected an IL-1β concentration of 5 ng/ml to stimulate the chondrocytes for 24 h in subsequent experiments.

### Effect of PA on Chondrocyte Viability

The viability of chondrocytes administered with PA was measured using a CCK-8 kit (Figure 2B). PA (20 and 40 μM) presented an inhibitory effect on cell growth at 24 and 48 h, whereas concentrations below 20 μM PA did not affect cell viability. In addition, toluidine blue staining showed no obvious morphological change in chondrocytes treated with PA at concentrations of 2.5, 5, and 10 μM (Figures 2C–G). Thus, PA at concentrations of 2.5, 5, and 10 μM were applied in the following experiments.

### PA Suppressed COX2, iNOS, ADAMTS5, and MMPs (MMP1, MMP3, and MMP13) in IL-1β-Treated Chondrocytes

Previous investigations have indicated that inflammation is a leading pathogenic factors in the progression of OA (Pelletier et al., 2001); therefore, anti-inflammatory therapy plays a pivotal role in the OA treatment. Thus, we designed experiments to explore whether PA could reduce the IL-1β-stimulated inflammation. The western blotting results revealed that different concentrations of PA (2.5, 5, and 10 μM) decreased the levels of protein iNOS and COX2 significantly compared to the group only treated with IL-1β. In addition, the anti-inflammatory property of PA on chondrocytes treated with IL-1β was concentration dependent (Figures 2H–J).

Catabolism is essential for keeping the homeostasis of the extracellular matrix (ECM). Among the catabolic indicators, ADAMTS5 and MMPs are the key enzymes related to chondrocyte catabolism; therefore, they were used to clarify the function of PA on IL-1β-treated cells. We observed that IL-1β (5 ng/ml) trigger high protein expression of ADAMTS5 and MMPs (MMP1, MMP3, and MMP13). However, PA reversed these trends shown in Figures 3A–E. The influence of PA on the degradation of cartilage ECM was also verified by RT-PCR according to mRNA expression (Figures 3G–I). As shown in Figure 3F, immunofluorescence analysis showed increased expression of MMP13 in the cytoplasm when cartilage cells were administration with IL-1β. However, PA (10 μM) decreased the protein expression of MMP13.

### PA Upregulated Collagen II, Aggrecan, and SOX9 in IL-1β-Induced Chondrocytes

**FIGURE 4:**
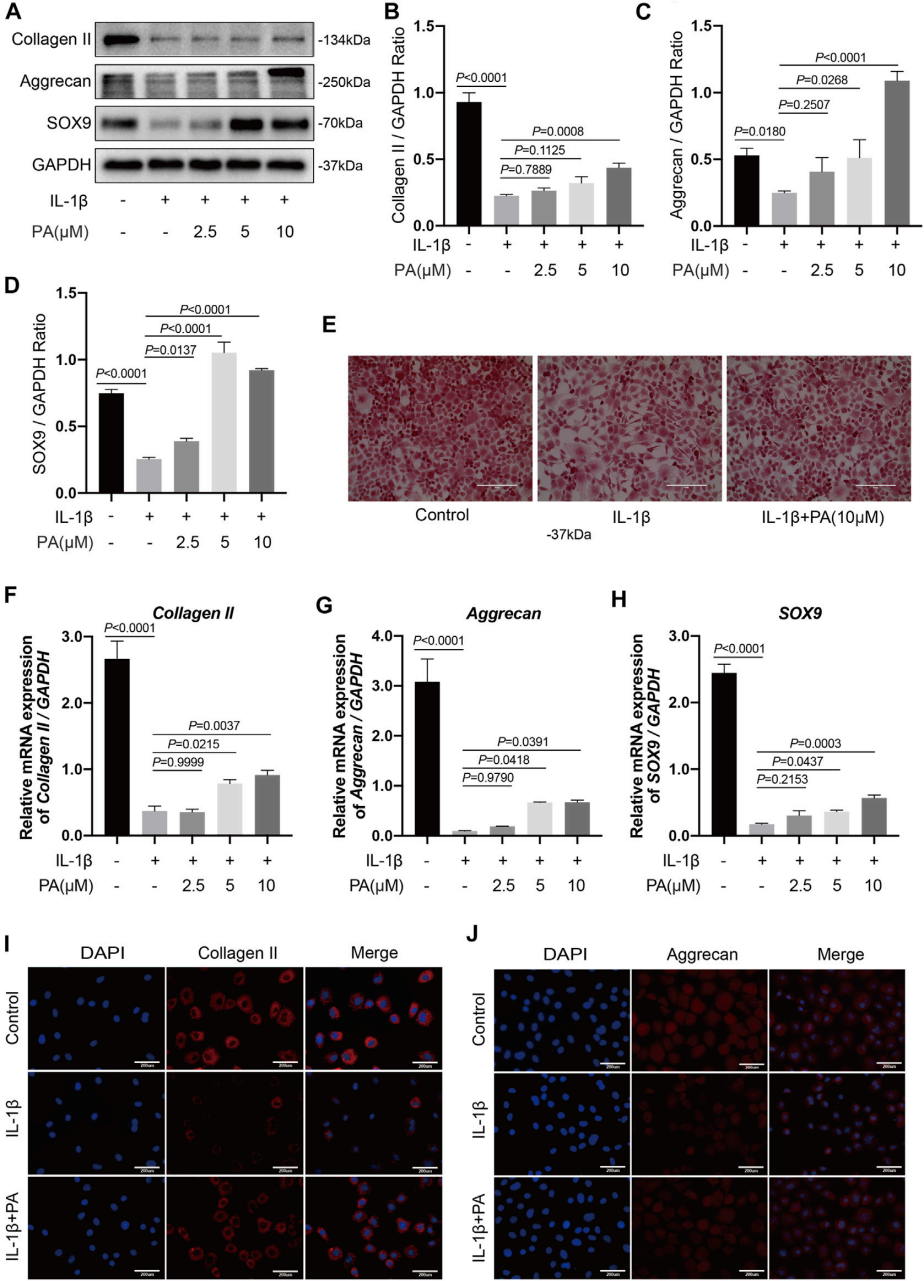
PA upregulated anabolic indicators including collagen II, aggrecan, and SOX9 expression in IL-1β-induced chondrocytes. **(A)** Western blotting results and **(B–D)** quantitative analysis of collagen II, aggrecan, and SOX9. **(E)** Safranin O staining reflected the content of proteoglycan among the control, IL-1β (5 ng/ml), and IL-1β (5 ng/ml) + PA (10 μM) groups (scale bar 200 μm). **(F–H)** Relative mRNA levels of collagen II, aggrecan, and SOX9 in chondrocytes stimulated with 5 ng/ml of IL-1β, alone or with PA (2.5, 5, and 10 μM) for 24 h. GAPDH was used as an internal reference. Data are presented as means ± SD (*n* = 3). The exact *p* value was marked in the corresponding figure and *p* < 0.05 was considered statistically significant. **(I,J)** Collagen II and aggrecan expression observed by immunofluorescence staining when chondrocytes were treated with 5 ng/ml of IL-1β, alone or with 10 μM of PA (scale bar 200 μm).

Similar to catabolism, anabolism is also a crucial factor in keeping the homeostasis of cartilage ECM (Mobasheri et al., 2017). Collagen II, aggrecan, and SOX9 are the most important proteins in the process of anabolism, and are beneficial for the metabolism of cartilage cells (Orhan et al., 2021); thus, we analyzed these above proteins. The data revealed that IL-1β could weaken the production of collagen II, aggrecan, and SOX9 in chondrocytes. However, this suppression was alleviated by the application of various concentrations of PA (Figures 4A–D), and the mRNA levels of collagen II, aggrecan, and SOX9 were confirmed by RT-qPCR analysis (Figures 4F–H). In addition, immunofluorescence analysis revealed reduced expression of collagen II and aggrecan when the cells were stimulated with IL-1β. However, PA (10 μM) upregulated the two proteins’ production (Figures 4I,J). Additionally, safranin O staining showed that the degree of redness, which reflects the proteoglycan content, was lower in the group pretreated with IL-1β than in the control group, but slightly enhanced after the administration of PA (Figure 4E); these results were consistent with those of western blotting and immunofluorescence analysis.

### PA Inhibited the IL-1β-Induced Activation of MAPK and NF-κB Signaling Pathways

**FIGURE 5:**
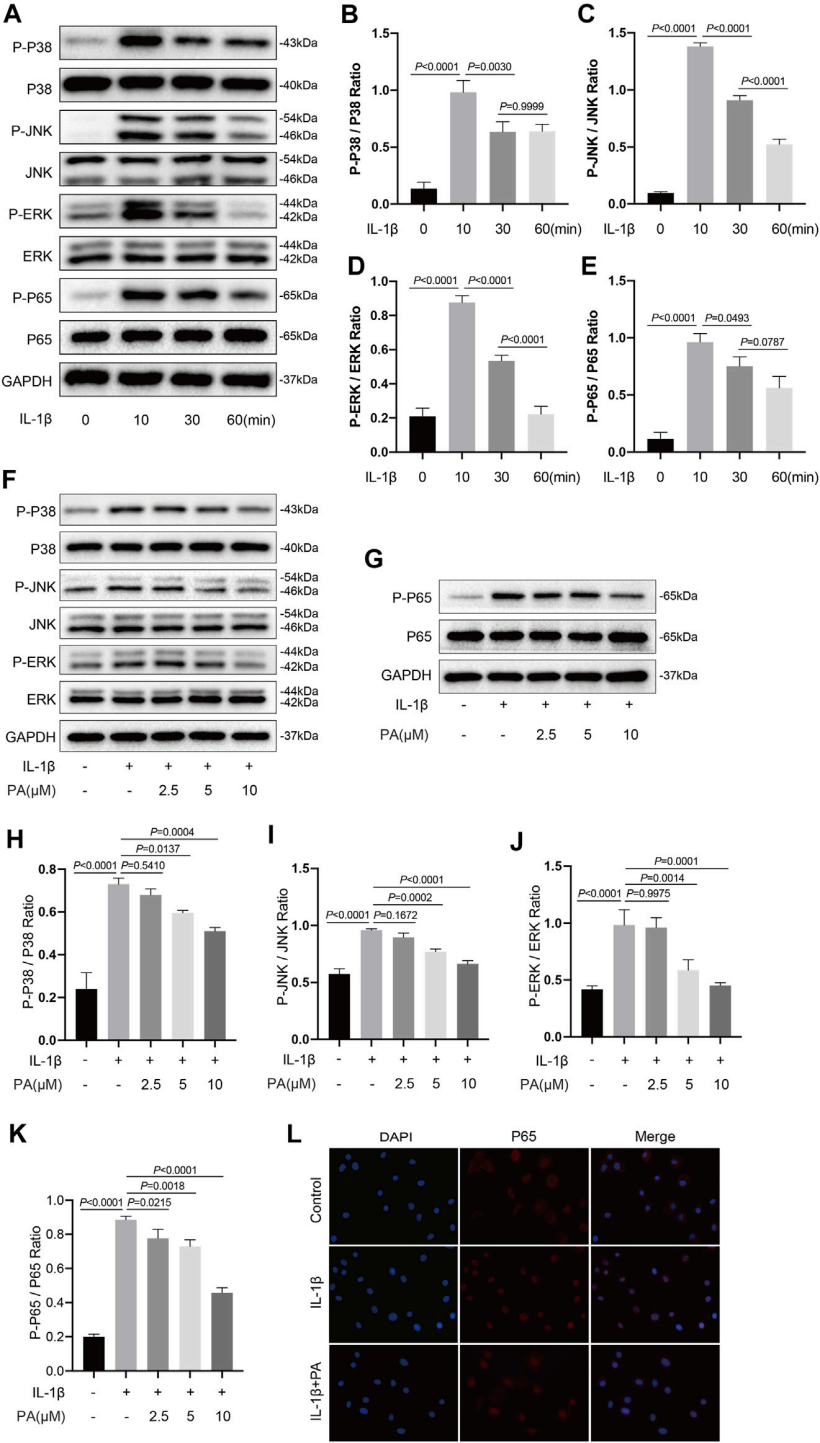
PA inhibited the IL-1β-induced activation of MAPK and NF-κB signaling pathways. **(A)** Western blots and **(B–E)** quantitative analysis of MAPK pathway related proteins (P-P38, P38, P-JNK, JNK, P-ERK, and ERK) and NF-κB pathway related proteins (P-P65, P65) in IL-1β-induced chondrocytes at the time points (0, 10, 30, and 60 min). **(F,G)** Western blotting results and **(H–K)** quantification analysis of MAPK pathway related proteins (P-P38, P38, P-JNK, JNK, P-ERK, and ERK) and NF-κB pathway related proteins (P-P65, P65) in chondrocytes pretreated with the administration of PA (2.5, 5, and 10 μM) for 24 h and followed the stimulation of 5 ng/ml of IL-1β for 10 min. **(L)** Nuclear translocation of P65 was detected by immunofluorescence staining after chondrocytes pretreated with the administration of PA (10 μM) for 24 h and followed the stimulation of 5 ng/ml of IL-1β for 10 min (scale bar 200 μm). Non-phosphorylated protein (P38, JNK, ERK, and P65) was used as an internal control. Data are presented as means ± SD (*n* = 3) and the exact *p* value was marked in the corresponding figure and *p* < 0.05 was considered statistically significant.

The transduction of MAPK and NF-κB pathways is an early event that promotes the development of OA. IL-1β, a cytokine, was widely used to activate the above two signaling pathways. According to current research, inhibiting the transduction of the above two signaling pathways triggered by IL-1β can alleviate the cartilage destruction in OA (Wang et al., 2021a). Thus, we analyzed proteins related to the MAPK and NF-κB pathways to determine the timing of activation of the above two pathways induced by IL-1β at those time points (0, 10, 30, and 60 min). Figures 5A–E shows that the IL-1β-induced activation of both MAPK and NF-κB pathways was time-dependent, with the activation peak occurring after 10 min of stimulation, before gradually weakening. Thus, we selected the strongest stimulus time point of 10 min to analyze the effect of PA on the two pathways. As demonstrated in Figures 5F–K, protein expression in the MAPK pathway (P-P38, P-JNK, P-ERK) and NF-κB pathway (P-P65) was significantly activated upon IL-1β treatment. In addition, our data (Figure 5L) demonstrated that P65 was localized in the cytoplasm of normal chondrocytes, while the tendency of p65 to transfer to the nucleus was increased after chondrocytes treated with IL-1β. However, the increased protein production of P-P38, P-JNK, P-ERK, P-P65, and increased transfer to the nucleus of P65 could be reversed by the application of PA.

### Knockdown of Integrin αVβ3 Weakened the Anti-inflammatory Effect of PA on IL-1β-Induced Chondrocytes

**FIGURE 6:**
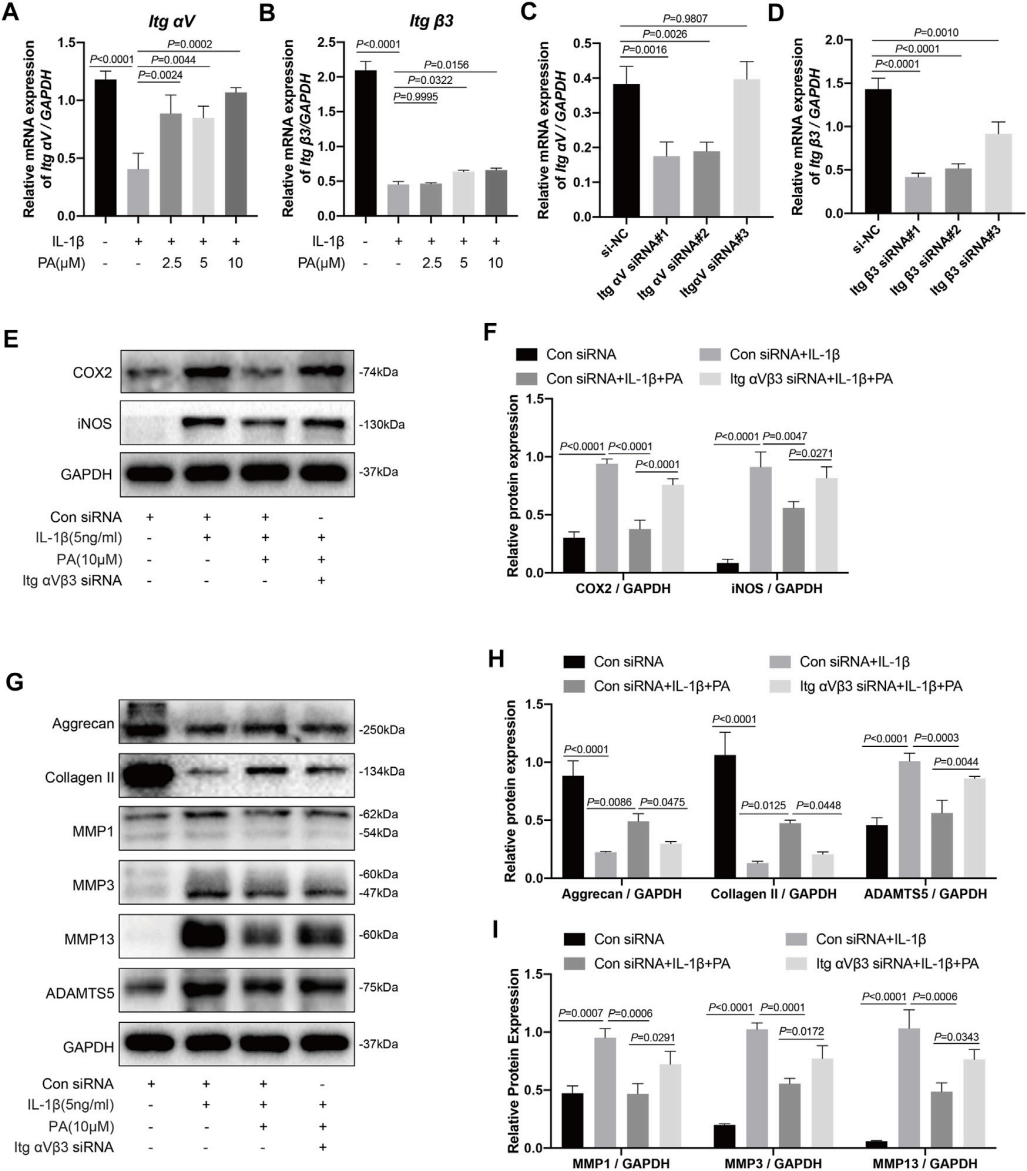
Knockdown of integrin αVβ3 weakened the anti-inflammatory, anabolism enhancing, and catabolism inhibiting effect of PA on IL-1β-induced chondrocytes. **(A,B)** Relative mRNA levels of integrin αV (Itg αV) and integrin β3 (Itg β3) in chondrocytes stimulated with 5 ng/ml of IL-1β, alone or with PA (2.5, 5, and 10 μM) for 24 h **(C,D)** Itg αV and Itg β3 were knocked down by siRNA transfection, and the knockdown efficiency was verified by RT-PCR. **(E,F)** Inflammatory markers (COX2, iNOS) were detected by western blotting and the band density of protein levels were quantified after mice chondrocytes were added with or without 5 ng/ml of IL-1β, 10 μM of PA, and Itg αVβ3 siRNA. **(G–I)** Western blotting was applied to measure the anabolic (aggrecan, collagen II) and catabolic markers (MMP1, MMP3, MMP13, and ADAMTS5) in the Itg αVβ3-deficiency mice chondrocytes along with or without the administration of 5 ng/ml of IL-1β and 10 μM of PA, and the band density of these protein levels were quantified in the histogram. GAPDH was used as an internal reference. Data are presented as means ± SD (*n* = 3). The exact *p* value was marked in the corresponding figure and *p* < 0.05 was considered statistically significant.

RT-qPCR detection revealed that the integrin αV/β3 (Itg αV/β3) levels in chondrocytes treated with IL-1β were less than those in untreated chondrocytes; however, this trend was reversed when chondrocytes were treated with PA (Figures 6A,B). Knockdown of integrin αV/β3 was then conducted by siRNA transfection, whose efficiency was verified by RT-qPCR; we then chose Itg αV siRNA#1 and Itg β3 siRNA#1 for follow-up experiments (Figures 6C,D). Integrin αVβ3 in the chondrocytes were knockout by using the above method, and these chondrocytes were followed the administration with 5 ng/ml IL-1β or 10 μM PA. After that, these pretreated chondrocytes were collected to detect protein expression by western blotting. We found that the inflammatory markers iNOS and COX2 were highly expressed in the IL-1β-treated group, and PA restrained the production of these two proteins; however, the inhibitory effect of PA on chondrocytes could be reversed when integrin αVβ3 was knocked down (Figures 6E,F).

### Knockdown of Integrin αVβ3 Reversed the Anabolism Enhancing/Catabolism Inhibiting Effect of PA on IL-1β-Induced Chondrocytes

In addition to the detection of inflammatory proteins in integrin αVβ3-deficient chondrocytes, we also measured the anabolic and catabolic indicators of chondrocytes. According to the results (Figures 6G–I), the production of anabolic-related proteins (aggrecan and collagen II) was decreased after chondrocytes stimulated with IL-1β. Moreover, PA upregulated these proteins, demonstrating the ability to enhance anabolism, whereas the expression level of these proteins was decreased again in cells deficient in integrin αVβ3. Furthermore, proteins related to the catabolism of chondrocytes, including ADAMTS5, MMP1, MMP3, and MMP13 were highly expressed after IL-1β treatment, and PA again exhibited the role of decreasing the production of these proteins. Moreover, the inhibitory effect of PA on catabolism could be reversed when integrin αVβ3 was knocked down in chondrocytes.

### Knockdown of Integrin αVβ3 Reversed the Inhibition of MAPK and NF-κB Signaling Pathways by PA

**FIGURE 7:**
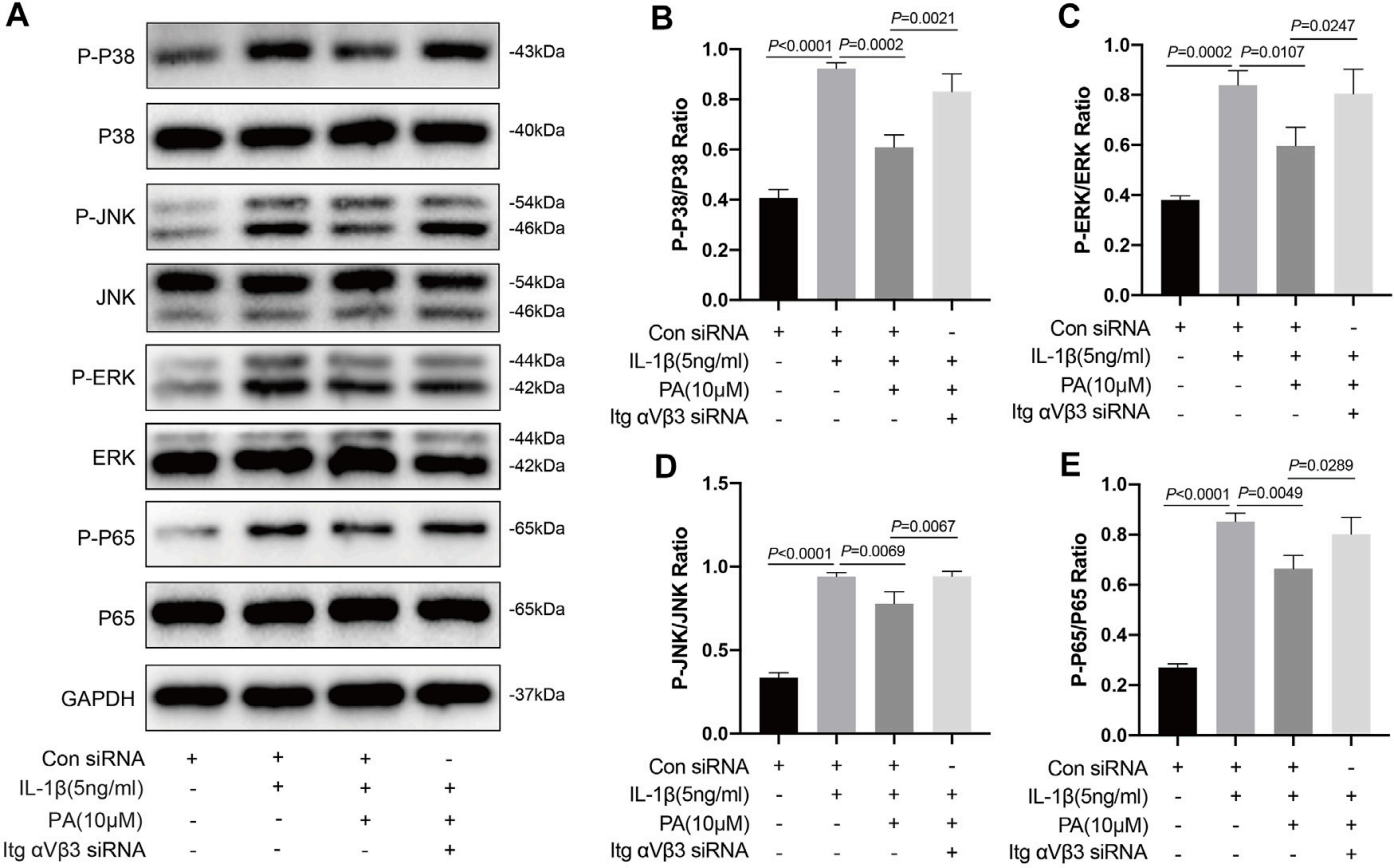
Knockdown of integrin αVβ3 reversed the inhibition of MAPK and NF-κB signaling pathways by PA. **(A)** Western blotting results and **(B–E)** quantitative analysis of MAPK pathway related proteins (P-P38, P38, P-JNK, JNK, P-ERK, and ERK) and NF-κB pathway related proteins (P-P65, P65) from the Itg αVβ3-deficiency mice chondrocytes along with or without the administration of 5 ng/ml of IL-1β and 10 μM of PA. The non-phosphorylated protein (P38, JNK, ERK, and P65) was used as an internal control. Data are presented as means ± SD (*n* = 3) The exact *p* value was marked in the corresponding figure and *p* < 0.05 was considered statistically significant.

Based on the above results, IL-1β can activate the MAPK and NF-κB pathways, which can trigger chondrocyte inflammation, enhance chondrocyte catabolism, inhibit chondrocyte anabolism, and lead to the destruction of cartilage and progression of OA. Conversely, PA can inhibit the activation of these two pathways. Thus, we explored whether PA would continue to affect the two pathways after integrin αVβ3 knockdown. According to Figure 7, compared to the control group, proteins in chondrocytes such as P-P38, P-JNK, P-ERK, and P-P65 were highly expressed in the IL-1β-treated group, but expressed at low levels after the application of PA. However, the decreased expression of these proteins could be reversed by the application of siRNA for integrin αVβ3 knockdown.

### *In Vivo* Attenuation of Cartilage Damage in Mice OA Model by PA

**FIGURE 8:**
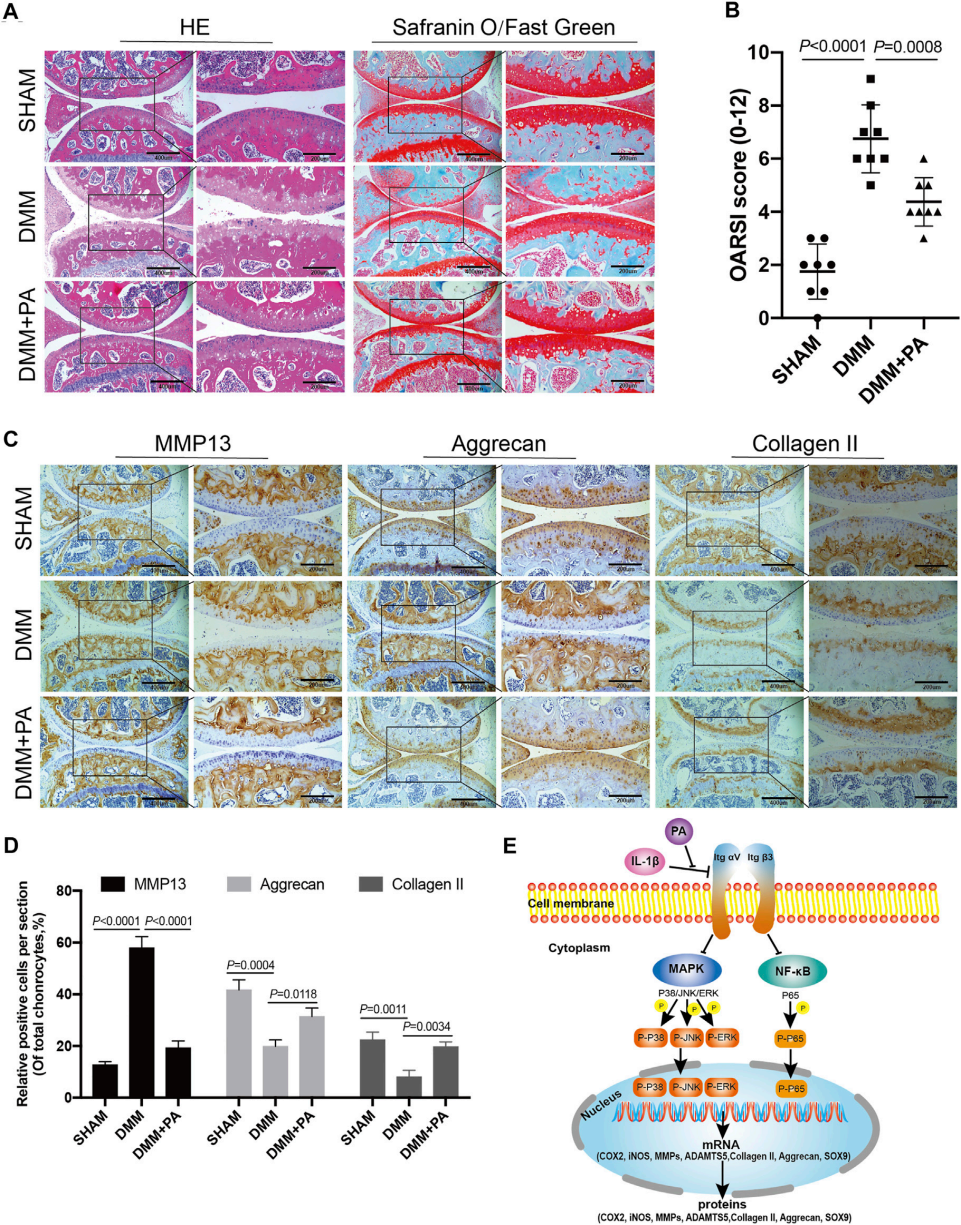
PA attenuated cartilage destruction in the *in vivo* mouse OA model. **(A)** HE, Safranin O, and Fast Green staining and **(B)** OARSI scores of mice knee joints from the sham, DMM, and DMM + PA groups at 8 weeks after the corresponding treatment (scale bars, 200 and 400 μm). **(C)** Immunohistochemistry staining and **(D)** quantification of the expression of MMP13, aggrecan, collagen II were measured among the three groups (scale bars 200 and 400 μm). Data are presented as means ± SD (*n* = 6). The exact *p* value was marked in the corresponding figure and *p* < 0.05 was considered statistically significant. **(E)** The potential molecular mechanism of PA’s chondroprotective effect. PA could alleviate the inflammatory response and cartilage degradation of IL-1β-induced chondrocytes by inhibting the MAPK and NF-κB signaling pathways, and the beneficial effect of PA on OA may be mediated through integrin αVβ3.

HE, safranin O/fast green, and immunohistological staining methods were applied to observe the changes among the aforementioned three groups. As shown in Figure 8A, compared to the sham group, obvious erosion of superficial articular cartilage and loss of proteoglycan were discovered in the DMM group. However, this adverse effect could be reversed by PA, as the DMM + PA group exhibited less cartilage destruction and richer proteoglycan than the DMM group. In addition, immunohistochemical staining showed consistent expression of MMP13, aggrecan, and collagen II among the three groups and with the *in vitro* experiment, with PA decreasing MMP13 production and increasing collagen II and aggrecan production in the *in vivo* mouse OA model (Figures 8C,D). Moreover, the OARSI score also indicated that injecting PA into the knee joint cavity could ameliorate the progression of OA in the mouse model (Figure 8B).

## Discussion

OA is a debilitating disease which placing a growing economic burden on society, accounting for almost 2.5% of the GDP in developed countries (Hiligsmann et al., 2013). However, the specific mechanism of OA remains unclear (Glyn-Jones et al., 2015; Jeon et al., 2017). Previous research revealed that inflammatory factors play a major part in the process of OA progression, and the balance of pro-inflammatory and anti-inflammatory cytokines is also essential for maintaining the steady state of catabolism and anabolism in articular cartilage (Sellam and Berenbaum, 2010; Lee et al., 2021). IL-1β, one main pro-inflammatory factor involved in the pathophysiology of OA, is often used as a stimulant for OA chondrocyte models because it can weaken cartilage anabolism and enhance cartilage catabolism, which is similar to the pathology of OA (Liacini et al., 2002; Huang et al., 2019). In other words, IL-1β triggers the high expression of matrix-degrading enzymes such as ADAMTS5, MMP1, MMP3, and MMP13, as well as the reduced synthesis of anabolic enzymes such as collagen II and aggrecan (Hosseinzadeh et al., 2016). These enzymes regulate the catabolism and anabolism of chondrocytes, and their imbalance will cause changes in the composition of the ECM. Thus, strategies to reduce the level of inflammation have proven to be effective for counteracting the disordered metabolic process of OA (Kapoor et al., 2011; Appleton, 2018; Lin et al., 2021). Furthermore, iNOS and COX2 are two pro-inflammatory mediators that play major roles in the pathogenesis of OA (Sellam and Berenbaum, 2010). COX2 typically exists in small amounts in vascular endothelial cells and catalyzes the formation of protective prostaglandin I_2_. However, under the stimulation of inflammation, cells such as monocytes, macrophages, and fibroblasts express COX2 in large amounts, leading to inflammation and pain (Jiang et al., 2020; Schjerning et al., 2020). High expression of iNOS and COX2 could further induce the production of catabolic factors, including ADAMTS5, MMP1, MMP3, and MMP13, which may lead to damage of the articular cartilage (Lepetsos et al., 2019). Strategies for reducing the level of inflammation have proven to be effective in treating OA (Matsumoto et al., 2021; Zeng et al., 2021). In this study, PA significantly reduced the generation of iNOS and COX2 in chondrocytes pretreated with IL-1β. In addition, the downregulation of catabolic enzymes including ADAMTS5, MMP1, MMP3, and MMP13 was also observed in IL-1β-induced chondrocytes.

The articular cartilage is an important part of the joint. It is smooth and elastic and can absorb and buffer stress to the maximum extent (Maki et al., 2021). After articular cartilage injury, the force absorption effect decreases, resulting in the progressive deterioration of joint performance (Elder and Athanasiou, 2009). An imbalance in the anabolism and catabolism of chondrocytes contributes to reduced force absorption (Chen et al., 2021; Tao et al., 2021). Collagen II, as one of the most important indexes of anabolism, is the main component of cartilage protein and provides a network structure that can obtain stability from other collagen types and non-collagen proteins and impart tensile strength to articular cartilage (Miao et al., 2021). In addition, aggrecan, another major anabolic indicator and cartilage protein, can incorporate water molecules into cartilage to provide compressive strength (Buckwalter et al., 2005; Pearle et al., 2005). The reduction of protein collagen II and aggrecan, which alters the ECM composition and generates tremendous changes in the mechanical environment of the cells in the cartilage matrix, is one of the crucial factors causing cartilage degradation in OA pathology (Maldonado and Nam, 2013). As shown by our results, the decreased generation of cartilage anabolic enzymes (including collagen II, aggrecan, and SOX9) induced by IL-1β could be reversed by PA. This indicates that PA promotes anti-inflammation, inhibits catabolism, and enhances anabolism in chondrocytes. Moreover, this type of cartilage protection characteristic was verified by the animal experiments, as intra-articular injection of PA attenuated cartilage damage in the *in vivo* mouse OA model.

The MAPK pathway, which includes the P38, JNK, and ERK pathways, is an important transmitter of signals from the surface of the cell to the inside of the nucleus (Sondergaard et al., 2010). The NF-κB pathway is a family of transcription factors. Both are ubiquitous signaling pathways that mediate cell proliferation, differentiation, and inflammatory response (Rigoglou and Papavassiliou, 2013); the signal transduction nodes among these pathways have become targets of related drugs development (Qi and Elion, 2005). In addition, MAPK and NF-κB pathways are essential for the progression of OA (Saklatvala, 2007; Herrero-Beaumont et al., 2019). As an upstream signaling pathway, the activation of MAPK signaling could contribute to the generation of aggrecanases and MMPs, which results in the degradation of the cartilage matrix (Sondergaard et al., 2010). P65, a key sub-unit of the NF-κB pathway, regulates the production of ADAMTS5 (Kobayashi et al., 2013) and SOX9 (Ushita et al., 2009). Furthermore, activation of the NF-κB pathway can trigger high expression of MMPs along with inflammation-related genes, such as iNOS and COX2, which are detrimental to chondrocytes (Clancy et al., 2001; Marcu et al., 2010). Therefore, targeting MAPK and NF-κB is beneficial to the maintenance of joint homeostasis. Our research revealed that IL-1β could significantly upregulate the expression of P-P38, P-JNK, P-ERK, and P-P65. However, upregulation of these proteins could be reversed by the application of PA. Thus, a chondroprotective effect of PA on OA was achieved by inhibiting MAPK and NF-κB signal transduction.

Integrin receptors are a family of transmembrane heterodimeric proteins that predominantly comprise α and β subunits. As one of the most common receptor families on cell surfaces, they can bind intracellular proteins and ECM proteins, such as fibronectin and type II and type VI collagens, and have the ability to modulate the proliferation, differentiation, and matrix remodeling of cells (Loeser, 2014; Tian et al., 2015; Kalli et al., 2017; Wang et al., 2017). Previous research suggested that integrin αVβ3 is an essential component of osteocytes because blocking integrin αvβ3 destroyed the morphology of osteocytes (Haugh et al., 2015). However, the role of integrin αvβ3 in chondrocytes remains controversial. Researchers have revealed that integrin αVβ3 can be found in normal adult articular chondrocytes (Loeser, 2014). Moreover, some studies have shown that integrin αVβ3 is highly expressed in osteoarthritic chondrocytes, exhibiting a cartilage damage effect, as the stimulation of integrin αVβ3 contributes to catabolic signaling, which results in matrix degradation, inflammatory response, cartilage breakdown, and progression of OA (Wang et al., 2019). Conversely, one study revealed that stimulation of integrin αVβ3 could restrain the protein expression of IL-1β and NO in chondrocytes and exert chondroprotective effects (Attur et al., 2000). In previous *in vivo* experiments, knockdown of the integrin α1 in mice led to the early appearance of OA (Zemmyo et al., 2003). Other researchers have shown that integrin αVβ3 can act as an intermediary that protects chondrocytes from apoptosis (Wang et al., 2018; Cheng et al., 2021). Moreover, MAPK regulates signaling pathways not only through various cytokines but also by the mechanical effects of integrins (Roca-Cusachs et al., 2012). MAPK and chondrocyte integrins are essential for the regulation of chondrocyte proliferation and matrix-degrading enzymes, including ADAMTS5 and MMPs, when dealing with mechanical stimuli (Perera et al., 2010; Liang et al., 2013). In our study, we found that integrin αVβ3 was expressed at low levels in chondrocytes with IL-1β treatment. However, PA upregulated the expression of integrin αVβ3 compared with the IL-1β treatment. We further explored whether the knockdown of integrin αVβ3 affects the inflammatory response, catabolism, anabolism of chondrocytes, and MAPK and NF-κB pathways under the administration of PA. Our data demonstrated that the knockdown of integrin αVβ3 weakened the anti-inflammatory effect of PA on IL-1β-induced chondrocytes. Simultaneously, it can reverse the anabolism enhancement/catabolism suppression effect of PA on IL-1β-stimulated chondrocytes. Additionally, integrin αVβ3 deficiency attenuated the inhibition of MAPK and NF-κB pathways by PA. Thus, we conclude that integrin αVβ3 may serve as a protective factor for IL-1β-stimulated chondrocytes, which is consistent with the results of previous research (Attur et al., 2000; Wang et al., 2018). As shown in Figure 8E, the anti-inflammatory, anabolism enhancing, catabolism inhibiting, and MAPK and NF-κB pathways inhibiting properties of PA on IL-1β-induced chondrocytes may be mediated through integrin αVβ3. We speculate that this may represent a feedback mechanism or an unknown relationship between integrin αVβ3 and PA. Thus, the detailed and complete mechanism behind the chondroprotective effect of PA and integrin αVβ3 on OA and the *in vivo* effect of integrin αVβ3 on arthritis requires further scrutiny.

In summary, our research is the first to demonstrate that PA plays a role in protecting articular cartilage through anti-inflammatory properties, anti-cartilage degradation, and MAPK and NF-κB signaling pathways inhibition *via in vivo* and *in vitro* experiments in mice. The chondroprotective effect of PA on OA may be mediated through integrin αVβ3. This indicates that PA or integrin αVβ3 might be a promising agent or molecular target for the treatment of OA.

## Data Availability

The original contributions presented in the study are included in the article/Supplementary Materials, further inquiries can be directed to the corresponding authors.

## References

[B1] Appleton C. T. (2018). Osteoarthritis Year in Review 2017: Biology. Osteoarthritis Cartilage 26 (3), 296–303. 10.1016/j.joca.2017.10.008 29061493

[B2] Attur M. G. Dave M. N. Clancy R. M. Patel I. R. Abramson S. B. Amin A. R. (2000). Functional Genomic Analysis in Arthritis-Affected Cartilage: Yin-Yang Regulation of Inflammatory Mediators by Alpha 5 Beta 1 and Alpha V Beta 3 Integrins. J. Immunol. 164 (5), 2684–2691. 10.4049/jimmunol.164.5.2684 10679109

[B3] Buckwalter J. A. Mankin H. J. Grodzinsky A. J. (2005). Articular Cartilage and Osteoarthritis. Instr. Course Lect 54, 465–480. 15952258

[B4] Chen D. H. Zheng G. Zhong X. Y. Lin Z. H. Yang S. W. Liu H. X. (2021). Oroxylin A Attenuates Osteoarthritis Progression by Dual Inhibition of Cell Inflammation and Hypertrophy. Food Funct. 12 (1), 328–339. 10.1039/d0fo02159h 33300913

[B5] Cheng C. Tian J. Zhang F. Deng Z. Tu M. Li L. (2021). WISP1 Protects against Chondrocyte Senescence and Apoptosis by Regulating αvβ3 and PI3K/Akt Pathway in Osteoarthritis. DNA Cel Biol 40 (4), 629–637. 10.1089/dna.2020.5926 33646053

[B6] Chuntakaruk H. Kongtawelert P. Pothacharoen P. (2021). Chondroprotective Effects of Purple Corn Anthocyanins on Advanced Glycation End Products Induction through Suppression of NF-Κb and MAPK Signaling. Sci. Rep. 11 (1), 1895. 10.1038/s41598-021-81384-4 33479339 PMC7820347

[B7] Clancy R. Rediske J. Koehne C. Stoyanovsky D. Amin A. Attur M. (2001). Activation of Stress-Activated Protein Kinase in Osteoarthritic Cartilage: Evidence for Nitric Oxide Dependence. Osteoarthritis Cartilage 9 (4), 294–299. 10.1053/joca.2000.0388 11399092

[B8] Crowley D. C. Lau F. C. Sharma P. Evans M. Guthrie N. Bagchi M. (2009). Safety and Efficacy of Undenatured Type II Collagen in the Treatment of Osteoarthritis of the Knee: a Clinical Trial. Int. J. Med. Sci. 6 (6), 312–321. 10.7150/ijms.6.312 19847319 PMC2764342

[B9] Deng Z. Hu X. Alahdal M. Liu J. Zhao Z. Chen X. (2021). High Expression of MAPK-14 Promoting the Death of Chondrocytes Is an Important Signal of Osteoarthritis Process. PeerJ 9, e10656. 10.7717/peerj.10656 33520453 PMC7812924

[B10] Elder B. D. Athanasiou K. A. (2009). Hydrostatic Pressure in Articular Cartilage Tissue Engineering: from Chondrocytes to Tissue Regeneration. Tissue Eng. Part. B Rev. 15 (1), 43–53. 10.1089/ten.teb.2008.0435 19196119 PMC2817666

[B11] Glyn-Jones S. Palmer A. J. Agricola R. Price A. J. Vincent T. L. Weinans H. (2015). Osteoarthritis. Lancet 386 (9991), 376–387. 10.1016/s0140-6736(14)60802-3 25748615

[B12] Han H. Qiu L. Wang X. Qiu F. Wong Y. Yao X. (2011). Physalins A and B Inhibit Androgen-independent Prostate Cancer Cell Growth through Activation of Cell Apoptosis and Downregulation of Androgen Receptor Expression. Biol. Pharm. Bull. 34 (10), 1584–1588. 10.1248/bpb.34.1584 21963499

[B13] Haugh M. G. Vaughan T. J. McNamara L. M. (2015). The Role of Integrin α(V)β(3) in Osteocyte Mechanotransduction. J. Mech. Behav. Biomed. Mater. 42, 67–75. 10.1016/j.jmbbm.2014.11.001 25460927

[B14] He H. Feng Y. S. Zang L. H. Liu W. W. Ding L. Q. Chen L. X. (2014). Nitric Oxide Induces Apoptosis and Autophagy; Autophagy Down-Regulates NO Synthesis in Physalin A-Treated A375-S2 Human Melanoma Cells. Food Chem. Toxicol. 71, 128–135. 10.1016/j.fct.2014.06.007 24952311

[B15] He H. Zang L. H. Feng Y. S. Chen L. X. Kang N. Tashiro S. (2013a). Physalin A Induces Apoptosis via P53-Noxa-Mediated ROS Generation, and Autophagy Plays a Protective Role against Apoptosis through P38-NF-Κb Survival Pathway in A375-S2 Cells. J. Ethnopharmacol 148 (2), 544–555. 10.1016/j.jep.2013.04.051 23684722

[B16] He H. Zang L. H. Feng Y. S. Wang J. Liu W. W. Chen L. X. (2013b). Physalin A Induces Apoptotic Cell Death and Protective Autophagy in HT1080 Human Fibrosarcoma Cells. J. Nat. Prod. 76 (5), 880–888. 10.1021/np400017k 23647462

[B17] Herrero-Beaumont G. Pérez-Baos S. Sánchez-Pernaute O. Roman-Blas J. A. Lamuedra A. Largo R. (2019). Targeting Chronic Innate Inflammatory Pathways, the Main Road to Prevention of Osteoarthritis Progression. Biochem. Pharmacol. 165, 24–32. 10.1016/j.bcp.2019.02.030 30825432

[B18] Hiligsmann M. Cooper C. Arden N. Boers M. Branco J. C. Luisa Brandi M. (2013). Health Economics in the Field of Osteoarthritis: an Expert's Consensus Paper from the European Society for Clinical and Economic Aspects of Osteoporosis and Osteoarthritis (ESCEO). Semin. Arthritis Rheum. 43 (3), 303–313. 10.1016/j.semarthrit.2013.07.003 23992801

[B19] Hosseinzadeh A. Kamrava S. K. Joghataei M. T. Darabi R. Shakeri-Zadeh A. Shahriari M. (2016). Apoptosis Signaling Pathways in Osteoarthritis and Possible Protective Role of Melatonin. J. Pineal Res. 61 (4), 411–425. 10.1111/jpi.12362 27555371

[B20] Huang X. Xi Y. Mao Z. Chu X. Zhang R. Ma X. (2019). Vanillic Acid Attenuates Cartilage Degeneration by Regulating the MAPK and PI3K/AKT/NF-κB Pathways. Eur. J. Pharmacol. 859, 172481. 10.1016/j.ejphar.2019.172481 31228458

[B21] Jeon O. H. Kim C. Laberge R. M. Demaria M. Rathod S. Vasserot A. P. (2017). Local Clearance of Senescent Cells Attenuates the Development of post-traumatic Osteoarthritis and Creates a Pro-regenerative Environment. Nat. Med. 23 (6), 775–781. 10.1038/nm.4324 28436958 PMC5785239

[B22] Jiang M. Deng H. Chen X. Lin Y. Xie X. Bo Z. (2020). The Efficacy and Safety of Selective COX-2 Inhibitors for Postoperative Pain Management in Patients after Total Knee/hip Arthroplasty: a Meta-Analysis. J. Orthop. Surg. Res. 15 (1), 39. 10.1186/s13018-020-1569-z 32024535 PMC7003344

[B23] Kalli A. C. Rog T. Vattulainen I. Campbell I. D. Sansom M. S. P. (2017). The Integrin Receptor in Biologically Relevant Bilayers: Insights from Molecular Dynamics Simulations. J. Membr. Biol. 250 (4), 337–351. 10.1007/s00232-016-9908-z 27465729 PMC5579164

[B24] Kang N. Jian J. F. Cao S. J. Zhang Q. Mao Y. W. Huang Y. Y. (2016). Physalin A Induces G2/M Phase Cell Cycle Arrest in Human Non-small Cell Lung Cancer Cells: Involvement of the P38 MAPK/ROS Pathway. Mol. Cel Biochem 415 (1-2), 145–155. 10.1007/s11010-016-2686-1 27000859

[B25] Kapoor M. Martel-Pelletier J. Lajeunesse D. Pelletier J. P. Fahmi H. (2011). Role of Proinflammatory Cytokines in the Pathophysiology of Osteoarthritis. Nat. Rev. Rheumatol. 7 (1), 33–42. 10.1038/nrrheum.2010.196 21119608

[B26] Kobayashi H. Hirata M. Saito T. Itoh S. Chung U. I. Kawaguchi H. (2013). Transcriptional Induction of ADAMTS5 Protein by Nuclear Factor-Κb (NF-Κb) Family Member RelA/p65 in Chondrocytes during Osteoarthritis Development. J. Biol. Chem. 288 (40), 28620–28629. 10.1074/jbc.M113.452169 23963448 PMC3789961

[B27] Lee W. Nims R. J. Savadipour A. Zhang Q. Leddy H. A. Liu F. (2021). Inflammatory Signaling Sensitizes Piezo1 Mechanotransduction in Articular Chondrocytes as a Pathogenic Feed-Forward Mechanism in Osteoarthritis. Proc. Natl. Acad. Sci. USA 118 (13), e2001611118. 10.1073/pnas.2001611118 33758095 PMC8020656

[B28] Lepetsos P. Papavassiliou K. A. Papavassiliou A. G. (2019). Redox and NF-Κb Signaling in Osteoarthritis. Free Radic. Biol. Med. 132, 90–100. 10.1016/j.freeradbiomed.2018.09.025 30236789

[B29] Li K. Zhang Y. Zhang Y. Jiang W. Shen J. Xu S. (2018). Tyrosine Kinase Fyn Promotes Osteoarthritis by Activating the β-catenin Pathway. Ann. Rheum. Dis. 77 (6), 935–943. 10.1136/annrheumdis-2017-212658 29555825

[B30] Liacini A. Sylvester J. Li W. Q. Zafarullah M. (2002). Inhibition of Interleukin-1-Stimulated MAP Kinases, Activating Protein-1 (AP-1) and Nuclear Factor Kappa B (NF-Kappa B) Transcription Factors Down-Regulates Matrix Metalloproteinase Gene Expression in Articular Chondrocytes. Matrix Biol. 21 (3), 251–262. 10.1016/s0945-053x(02)00007-0 12009331

[B31] Liang W. Ren K. Liu F. Cui W. Wang Q. Chen Z. (2013). Periodic Mechanical Stress Stimulates the FAK Mitogenic Signal in Rat Chondrocytes through ERK1/2 Activity. Cell Physiol Biochem 32 (4), 915–930. 10.1159/000354495 24217647

[B32] Lianxu C. Hongti J. Changlong Y. (2006). NF-κBp65-specific siRNA Inhibits Expression of Genes of COX-2, NOS-2 and MMP-9 in Rat IL-1β-induced and TNF-α-Induced Chondrocytes. Osteoarthritis and Cartilage 14 (4), 367–376. 10.1016/j.joca.2005.10.009 16376111

[B33] Lin Y. H. Hsiao Y. H. Ng K. L. Kuo Y. H. Lim Y. P. Hsieh W. T. (2020). Physalin A Attenuates Inflammation through Down-Regulating C-Jun NH2 Kinase phosphorylation/Activator Protein 1 Activation and Up-Regulating the Antioxidant Activity. Toxicol. Appl. Pharmacol. 402, 115115. 10.1016/j.taap.2020.115115 32634518

[B34] Lin Y. Y. Ko C. Y. Liu S. C. Wang Y. H. Hsu C. J. Tsai C. H. (2021). miR‐144‐3p Ameliorates the Progression of Osteoarthritis by Targeting IL‐1β: Potential Therapeutic Implications. J. Cel Physiol 236, 6988–7000. 10.1002/jcp.30361 33772768

[B35] Loeser R. F. (2014). Integrins and Chondrocyte-Matrix Interactions in Articular Cartilage. Matrix Biol. 39, 11–16. 10.1016/j.matbio.2014.08.007 25169886 PMC4699681

[B36] Maki K. Nava M. M. Villeneuve C. Chang M. Furukawa K. S. Ushida T. (2021). Hydrostatic Pressure Prevents Chondrocyte Differentiation through Heterochromatin Remodeling. J. Cel Sci 134 (2). 10.1242/jcs.247643 PMC7860130 33310912

[B37] Maldonado M. Nam J. (2013). The Role of Changes in Extracellular Matrix of Cartilage in the Presence of Inflammation on the Pathology of Osteoarthritis. Biomed. Res. Int. 2013, 284873. 10.1155/2013/284873 24069595 PMC3771246

[B38] Marcu K. B. Otero M. Olivotto E. Borzi R. M. Goldring M. B. (2010). NF-kappaB Signaling: Multiple Angles to Target OA. Curr. Drug Targets 11 (5), 599–613. 10.2174/138945010791011938 20199390 PMC3076145

[B39] Matsumoto Y. Shivappa N. Sugioka Y. Tada M. Okano T. Mamoto K. (2021). Change in Dietary Inflammatory index Score Is Associated with Control of Long-Term Rheumatoid Arthritis Disease Activity in a Japanese Cohort: the TOMORROW Study. Arthritis Res. Ther. 23 (1), 105. 10.1186/s13075-021-02478-y 33832530 PMC8028141

[B40] Miao X. Wu Y. Wang P. Zhang Q. Zhou C. Yu X. (2021). Vorinostat Ameliorates IL‐1α‐induced Reduction of Type II Collagen by Inhibiting the Expression of ELF3 in Chondrocytes. J. Biochem. Mol. Toxicol. 35, e22844. 10.1002/jbt.22844 34250664 PMC8519056

[B41] Mobasheri A. Rayman M. P. Gualillo O. Sellam J. van der Kraan P. Fearon U. (2017). The Role of Metabolism in the Pathogenesis of Osteoarthritis. Nat. Rev. Rheumatol. 13 (5), 302–311. 10.1038/nrrheum.2017.50 28381830

[B42] Orhan C. Juturu V. Sahin E. Tuzcu M. Ozercan I. H. Durmus A. S. (2021). Undenatured Type II Collagen Ameliorates Inflammatory Responses and Articular Cartilage Damage in the Rat Model of Osteoarthritis. Front. Vet. Sci. 8 (86), 617789. 10.3389/fvets.2021.617789 33748207 PMC7970046

[B43] Pearle A. D. Warren R. F. Rodeo S. A. (2005). Basic Science of Articular Cartilage and Osteoarthritis. Clin. Sports Med. 24 (1), 1–12. 10.1016/j.csm.2004.08.007 15636773

[B44] Pelletier J. P. Martel-Pelletier J. Abramson S. B. (2001). Osteoarthritis, an Inflammatory Disease: Potential Implication for the Selection of New Therapeutic Targets. Arthritis Rheum. 44 (6), 1237–1247. 10.1002/1529-0131 11407681

[B45] Perera P. M. Wypasek E. Madhavan S. Rath-Deschner B. Liu J. Nam J. (2010). Mechanical Signals Control SOX-9, VEGF, and C-Myc Expression and Cell Proliferation during Inflammation via Integrin-Linked Kinase, B-Raf, and ERK1/2-dependent Signaling in Articular Chondrocytes. Arthritis Res. Ther. 12 (3), R106. 10.1186/ar3039 20509944 PMC2911896

[B46] Petersen K. K. Olesen A. E. Simonsen O. Arendt-Nielsen L. (2019). Mechanistic Pain Profiling as a Tool to Predict the Efficacy of 3-week Nonsteroidal Anti-inflammatory Drugs Plus Paracetamol in Patients with Painful Knee Osteoarthritis. Pain 160 (2), 486–492. 10.1097/j.pain.0000000000001427 30371559

[B47] Qi M. Elion E. A. (2005). MAP Kinase Pathways. J. Cel Sci 118 (Pt 16), 3569–3572. 10.1242/jcs.02470 16105880

[B48] Rigoglou S. Papavassiliou A. G. (2013). The NF-Κb Signalling Pathway in Osteoarthritis. Int. J. Biochem. Cel Biol 45 (11), 2580–2584. 10.1016/j.biocel.2013.08.018 24004831

[B49] Roca-Cusachs P. Iskratsch T. Sheetz M. P. (2012). Finding the Weakest Link: Exploring Integrin-Mediated Mechanical Molecular Pathways. J. Cel Sci 125 (Pt 13), 3025–3038. 10.1242/jcs.095794 PMC6518164 22797926

[B50] Saklatvala J. (2007). Inflammatory Signaling in Cartilage: MAPK and NF-kappaB Pathways in Chondrocytes and the Use of Inhibitors for Research into Pathogenesis and Therapy of Osteoarthritis. Curr. Drug Targets 8 (2), 305–313. 10.2174/138945007779940115 17305508

[B51] Schjerning A. M. McGettigan P. Gislason G. (2020). Cardiovascular Effects and Safety of (Non-aspirin) NSAIDs. Nat. Rev. Cardiol. 17 (9), 574–584. 10.1038/s41569-020-0366-z 32322101

[B52] Schmitz N. Laverty S. Kraus V. B. Aigner T. (2010). Basic Methods in Histopathology of Joint Tissues. Osteoarthritis Cartilage 18 Suppl 3 (Suppl. 3), S113–S116. 10.1016/j.joca.2010.05.026 20864017

[B53] Sellam J. Berenbaum F. (2010). The Role of Synovitis in Pathophysiology and Clinical Symptoms of Osteoarthritis. Nat. Rev. Rheumatol. 6 (11), 625–635. 10.1038/nrrheum.2010.159 20924410

[B54] Shepard N. Mitchell N. (1976). Simultaneous Localization of Proteoglycan by Light and Electron Microscopy Using Toluidine Blue O. A Study of Epiphyseal Cartilage. J. Histochem. Cytochem. 24 (5), 621–629. 10.1177/24.5.132503 132503

[B55] Shin J. M. Lee K. M. Lee H. J. Yun J. H. Nho C. W. (2019). Physalin A Regulates the Nrf2 Pathway through ERK and P38 for Induction of Detoxifying Enzymes. BMC Complement. Altern. Med. 19 (1), 101. 10.1186/s12906-019-2511-y 31072358 PMC6507134

[B56] Sondergaard B. C. Schultz N. Madsen S. H. Bay-Jensen A. C. Kassem M. Karsdal M. A. (2010). MAPKs Are Essential Upstream Signaling Pathways in Proteolytic Cartilage Degradation-Ddivergence in Pathways Leading to Aggrecanase and MMP-Mediated Articular Cartilage Degradation. Osteoarthritis Cartilage 18 (3), 279–288. 10.1016/j.joca.2009.11.005 19932675

[B57] Tao S. C. Huang J. Y. Gao Y. Li Z. X. Wei Z. Y. Dawes H. (2021). Small Extracellular Vesicles in Combination with Sleep-Related circRNA3503: A Targeted Therapeutic Agent with Injectable Thermosensitive Hydrogel to Prevent Osteoarthritis. Bioact Mater. 6 (12), 4455–4469. 10.1016/j.bioactmat.2021.04.031 34027234 PMC8120802

[B58] Tian J. Zhang F. J. Lei G. H. (2015). Role of Integrins and Their Ligands in Osteoarthritic Cartilage. Rheumatol. Int. 35 (5), 787–798. 10.1007/s00296-014-3137-5 25261047

[B59] Ushita M. Saito T. Ikeda T. Yano F. Higashikawa A. Ogata N. (2009). Transcriptional Induction of SOX9 by NF-kappaB Family Member RelA in Chondrogenic Cells. Osteoarthritis Cartilage 17 (8), 1065–1075. 10.1016/j.joca.2009.02.003 19254740

[B60] Wang H. Jiang Z. Pang Z. Qi G. Hua B. Yan Z. (2021a). Engeletin Protects against TNF-α-Induced Apoptosis and Reactive Oxygen Species Generation in Chondrocytes and Alleviates Osteoarthritis *In Vivo* . J. Inflamm. Res. 14, 745–760. 10.2147/jir.S297166 33727849 PMC7955871

[B61] Wang K. K. Metlapally R. Wildsoet C. F. (2017). Expression Profile of the Integrin Receptor Subunits in the Guinea Pig Sclera. Curr. Eye Res. 42 (6), 857–863. 10.1080/02713683.2016.1262045 28094579 PMC5464978

[B62] Wang L. Gu J. Zong M. Zhang Q. Li H. Li D. (2021b). Anti-inflammatory Action of Physalin A by Blocking the Activation of NF-Κb Signaling Pathway. J. Ethnopharmacol 267, 113490. 10.1016/j.jep.2020.113490 33091501

[B63] Wang Q. Onuma K. Liu C. Wong H. Bloom M. S. Elliott E. E. (2019). Dysregulated Integrin αVβ3 and CD47 Signaling Promotes Joint Inflammation, Cartilage Breakdown, and Progression of Osteoarthritis. JCI Insight 4 (18). 10.1172/jci.insight.128616 PMC6795293 31534047

[B64] Wang Z. Boyko T. Tran M. C. LaRussa M. Bhatia N. Rashidi V. (2018). DEL1 Protects against Chondrocyte Apoptosis through Integrin Binding. J. Surg. Res. 231, 1–9. 10.1016/j.jss.2018.04.066 30278915

[B65] Woolf A. D. Pfleger B. (2003). Burden of Major Musculoskeletal Conditions. Bull. World Health Organ. 81 (9), 646–656. 10.1590/S0042-96862003000900007 14710506 PMC2572542

[B66] Wu Y. Wang Z. Fu X. Lin Z. Yu K. (2020). Geraniol-mediated Osteoarthritis Improvement by Down-Regulating PI3K/Akt/NF-Κb and MAPK Signals: *In Vivo* and *In Vitro* Studies. Int. Immunopharmacol 86, 106713. 10.1016/j.intimp.2020.106713 32590318

[B67] Zemmyo M. Meharra E. J. Kühn K. Creighton-Achermann L. Lotz M. (2003). Accelerated, Aging-dependent Development of Osteoarthritis in Alpha1 Integrin-Deficient Mice. Arthritis Rheum. 48 (10), 2873–2880. 10.1002/art.11246 14558093

[B68] Zeng C. Doherty M. Persson M. S. M. Yang Z. Sarmanova A. Zhang Y. (2021). Comparative Efficacy and Safety of Acetaminophen, Topical and Oral Non-steroidal Anti-inflammatory Drugs for Knee Osteoarthritis: Evidence from a Network Meta-Analysis of Randomized Controlled Trials and Real-World Data. Osteoarthritis and Cartilage 29, 1242–1251. 10.1016/j.joca.2021.06.004 34174454

