# Supplementary figures and images for: Physalin A Inhibits MAPK and NF-κB Signal Transduction Through Integrin αVβ3 and Exerts Chondroprotective Effect
Source: Front Pharmacol. 2021 Dec 1;12:761922. doi: 10.3389/fphar.2021.761922 (PMC8678602; doi:10.3389/fphar.2021.761922)

Relative mRNA expression  
of *Itg*  $\alpha$ V and *Itg*  $\beta$ 3

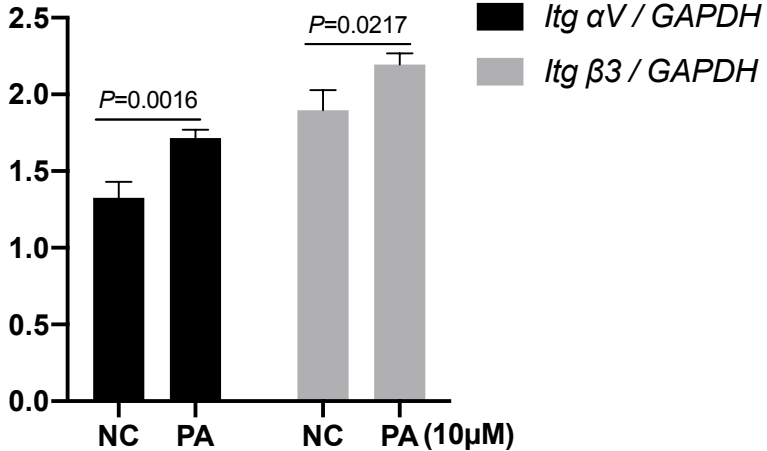

Supplement: Supplementary file 1 [file Image1.pdf]
